# Mechanical behavior of a titanium alloy scaffold mimicking trabecular structure

**DOI:** 10.1186/s13018-019-1489-y

**Published:** 2020-02-07

**Authors:** Chunqiu Zhang, Lan Zhang, Lu Liu, Linwei Lv, Lilan Gao, Nian Liu, Xin Wang, Jinduo Ye

**Affiliations:** 1 Tianjin Key Laboratory for Advanced Mechatronic System Design and Intelligent Control, National Demonstration Center for Experimental Mechanical and Electrical Engineering Education, Tianjin, University of Technology, Tianjin, 300384 People’s Republic of China; 2 Just Huajian Medical Device (Tianjin) Co., Ltd., Tianjin, 300190 China

**Keywords:** 3D printing, Unit cell, Trabecula bone, Porosity, Experimental research

## Abstract

**Background:**

Additively manufactured porous metallic structures have recently received great attention for bone implant applications. The morphological characteristics and mechanical behavior of 3D printed titanium alloy trabecular structure will affect the effects of artificial prosthesis replacement. However, the mechanical behavior of titanium alloy trabecular structure at present clinical usage still is lack of in-depth study from design to manufacture as well as from structure to mechanical function.

**Methods:**

A unit cell of titanium alloy was designed to mimick trabecular structure. The controlled microarchitecture refers to a repeating array of unit-cells, composed of titanium alloy, which make up the scaffold structure. Five kinds of unit cell mimicking trabecular structure with different pore sizes and porosity were obtained by modifying the strut sizes of the cell and scaling the cell as a whole. The titanium alloy trabecular structure was fabricated by 3D printing based on Electron Beam Melting (EBM). The paper characterized the difference between the designs and fabrication of trabecular structures, as well as mechanical properties and the progressive collapse behavior and failure mechanism of the scaffold.

**Results:**

The actual porosities of the EBM-produced bone trabeculae are lower than the designed, and the load capacity of a bearing is related to the porosity of the structure. The larger the porosity of the structure, the smaller the stiffness and the worse the load capacity is. The fracture interface of the trabecular structure under compression is at an angle of 45^o^ with respect to the compressive axis direction, which conforms to Tresca yield criterion. The trabeculae-mimicked unit cell is anisotropy. Under quasi-static loading, loading speed has no effect on mechanical performance of bone trabecular specimens. There is no difference of the mechanical performance at various orientations and sites in metallic workspace. The elastic modulus of the scaffold decreases by 96%–93% and strength reduction 96%–91%, compared with titanium alloy dense metals structure. The apparent elastic modulus of the unit-cell-repeated scaffold is 0.39–0.618 GPa, which is close to that of natural bone and stress shielding can be reduced.

**Conclusion:**

We have systematically studied the structural design, fabrication and mechanical behavior of a 3D printed titanium alloy scaffold mimicking trabecula bone. This study will be benefit of the application of prostheses with proper structures and functions.

## Introduction

At present, 3D printing technology has developed vigorously in the medical field [1, 2] and has been successfully used in orthopedic treatment [3, 4]. The 3D printing technology cannot only realize the individualized manufacture of medical devices, but also is superior to the traditional technology in the construction of the microstructures of implants [5, 6]. It can make titanium alloy [7, 8] and other metal powders into fabricate three-dimensional porous metal implants (metal trabeculae) with different porous structures, whose microstructure is similar to human bone trabecula, and whose porosity, pore size, pore volume, spatial arrangement and other surface properties as well as elastic modulus can be completely determined by design [9, 10]. The trabecular bone structure greatly improves the bioactivity of implants, it has high pore connectivity and is conducive to the adhesion, proliferation and differentiation of osteoblasts [11–13]. At the same time, it can guide bone tissue to grow into the pore and form implant-bone bio-fixation. The bonding strength with bone interface increases by about three times compared with that of dense metal implants [14–17]. In addition, the interconnected porous structure is conducive to the transport and circulation of body fluids and nutrients, speeding up tissue healing, and improving the biological stability of implants in the short term. With the development of 3D printing technology and new biological materials, the application of trabecular structure in artificial joint replacement [18] is increasing, and the design, fabrication and mechanical properties of trabecular structures are the focus of current research [19].

The morphological characteristics and mechanical behavior of 3D printed titanium alloy trabecular structure will affect the effects of artificial prosthesis replacement, therefore, the aspects need to be further researched. Wang Chunxiao [20] et al. designed and manufactured titanium alloy scaffolds with different pore structures using metal 3D printing technology, and observed its micro-pore characteristics and mechanical properties. Sakkadech Limmahakhun [21, 22] et al. taking the cylindrical octahedron structure as a unit, fabricated four kinds of porous structures with different pore size by Selective laser melting technology, and testing their mechanical properties and biological behavior. Li X [23] et al. studied the surface properties [24, 25], mechanical properties [26, 27], and its biological behavior [28, 29] of porous titanium alloy made by electron beam melting (EBM). However, the mechanical behavior of titanium alloy trabecular structure at present stage of clinical usage still lacks of in-depth study, from design to manufacture, from structure to mechanical function.

In this paper, 3D printed titanium alloy scaffold mimicking trabecular structure was fabricated by EBM. The differences between design and manufacture, the effects of different porosity and pore size on compressive mechanical properties and the mechanism of structural damage are studied.

## Materials and methods

### Fabrication of scaffold specimens

**Fig. 1:**
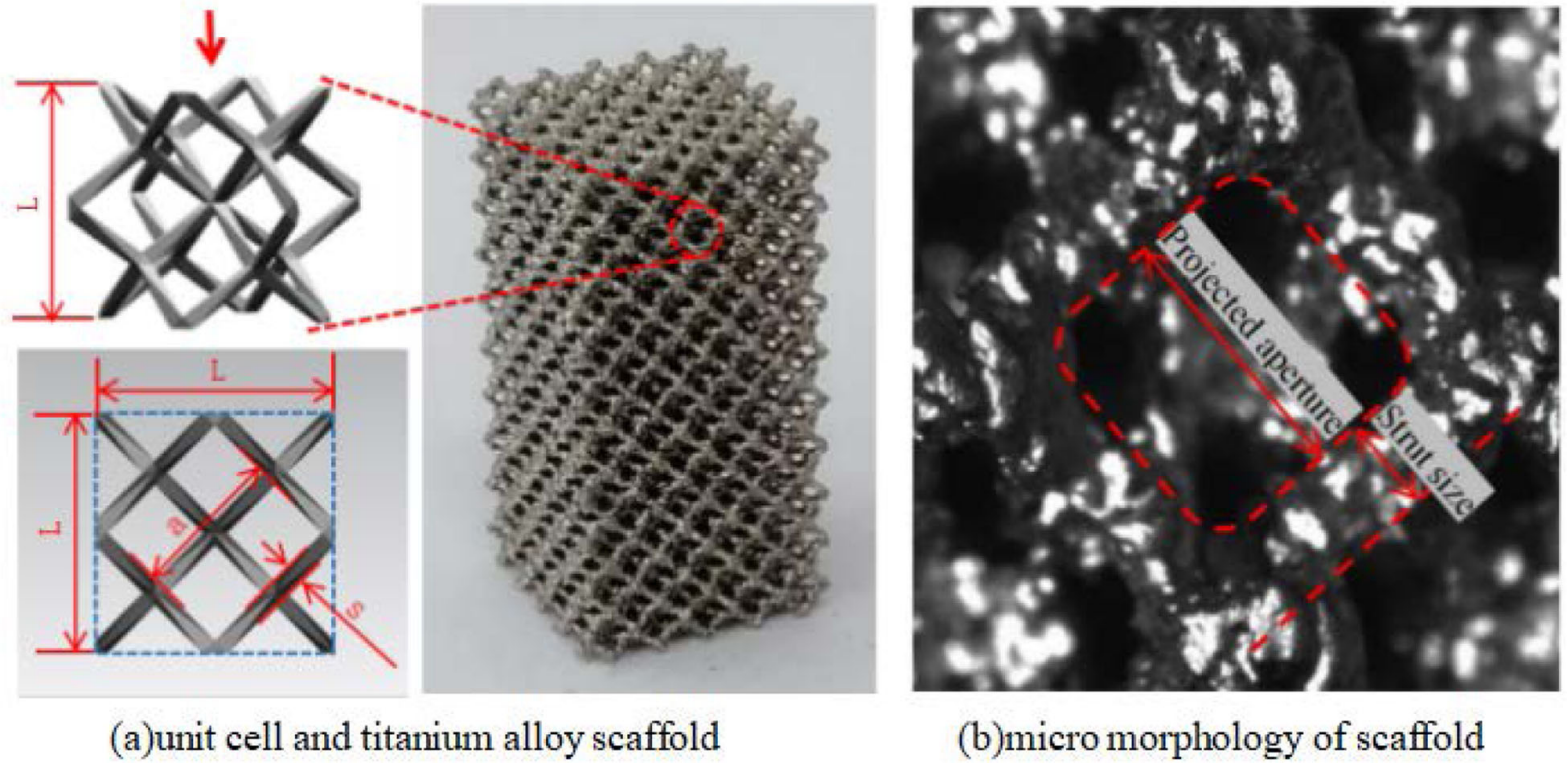
Titanium alloy scaffold built by unit cell (L, a and s are unit, projected aperture and strut size, respectively) and micro morphology of titanium alloy scaffolds mimicking bone trabeculae. **a** unit cell and titanium alloy scaffold, **b** micro morphology of scaffold

The overall structure and the building unit cells were selected, as shown in Fig. 1a. The unit cell called “Fine Strut 2.0”, as the original structure, which can be exactly contained in a square body with a side length of L = 2.0 mm, is rhombic and the strut size of that is 0.1467 mm. By scaling the original structure as a whole, that is to say, by changing the worth of “L” to obtain “Fine Strut 1.75” and “Fine Strut 2.5”. In addition, the strut size of the original structure increases to 0.2739 mm to obtain “Mid Strut 2.0”, then which is magnified 1.25 times as a whole to get “Mid Strut 2.5”. Five scaffolds with different pore sizes and porosities were obtained. Copy the unit cells respectively along the x, y and z axes, and then perform Boolean operations on them. Five kinds of cuboid scaffolds with cross section size of 10 *10 mm and height of 20 mm were established with these five cells as units, respectively. Cuboid scaffold specimens (Provided by Tianjin Just Huajian Medical Devices Co., Ltd.) were prepared by EBM with Ti6Al4V titanium alloy powder as raw material. The design dimensions are shown in Table 1.

**Table 1:** Design size of cellular structure and theoretical porosity corresponding of scaffold specimens

Cell specifications	L/mm	Strut size/mm	Aperture/mm	Projected aperture/mm	Specimen size/mm	Theoretical porosity of specimens/%
Fine Strut 1.75	1.75	0.1284	0.6571	1.1090	10 × 10 × 20	96.065
Fine Strut 2.0	2.0	0.1467	0.7511	1.2676	10 × 10 × 20	96.07
Fine Strut 2.5	2.5	0.1834	0.9388	1.5843	10 × 10 × 20	96.066
Mid Strut 2.0	2.0	0.2739	0.6615	1.1403	10 × 10 × 20	87.415
Mid Strut 2.5	2.5	0.3424	0.8269	1.4254	10 × 10 × 20	87.414

### Microstructural characterization

High temperature melting and solidification of metal powders can cause intracellular bending and ripple, resulting in geometric irregularity and uneven surface. The trabecular structure of titanium alloy under high magnifier is shown in Fig. 1b.

The strut size and aperture of scaffold specimens were measured by digital image correlation equipment (2D-DIC), including high magnifying lens, computer and data processing software. Measure the strut size and aperture of the image in the display window with vernier caliper. Measure three places and then take the average value. Finally divide the magnification factor to calculate the actual size.

The projection of scaffold specimens in the vertical direction can only be observed under a high magnifying glass. The observed “pore” is not the real pore of the scaffold, but its projected pore in the vertical direction. For ease of comparison, both the scaffold model and the manufactured scaffold specimens measured the aperture and strut size under projection.

Dense cylindrical titanium alloy specimens also were fabricated by 3D printing. The diameter of cross section is 20 mm, and the height of specimen is 113 mm. The volume of the specimen was measured by vernier caliper and the mass of that was obtained by high precision balance weighing. Finally the density of 3D printed dense titanium alloy material was obtained by volume and mass calculation. This density is used to calculate the actual porosity of 3D printed titanium alloy scaffold specimens.

### Mechanical tests

Quasi-static uniaxial compression tests were carried out on the fabricated scaffold specimens using the electronic universal testing machine (Changchun Kexin Test Instrument Co., Ltd. Type WDW-10).

The correlation between porosity and mechanical properties were studied on scaffold specimens with different aperture and porosity at the same loading speed. The compression tests included researching the anisotropy of specimens under the same loading speed from two different loading directions as well as the effects of different loading speeds on mechanical properties. Moreover, the mechanical properties of specimens taken from different 9 locations and 3 orientations of the 3D printer’ working space were studied.

### Data analysis

The experimental data from the mechanical test was analyzed. Descriptive statistics were used to summarize sample characteristics and our primary variables of interest. All data analysis is done in Matlab. A one-way analysis of variance (ANOVA) was carried out to determine the statistical variances among the contact pressure, peak contact pressure and contact area of different loading rates and different loads for the intact samples and defect samples, respectively. Test data used in the figures represented mean values, while the standard errors above and below mean values were indicated by error bars.

## Results

### Microstructural characterization

The density of 3D printed dense titanium alloy material is 4.3541037 g/cm^3^ calculated from 3D printed dense cylindrical specimens. The actual strut size, aperture and porosity of the prepared scaffold specimens are shown in Table 2.

**Table 2:** Actual strut size, aperture and porosity of five different specifications of scaffold specimens

Specimen specifications	Strut size/mm	Projected aperture/mm	Actual porosity/%
Fine Strut 1.75	0.4	0.766667	61.444
Fine Strut 2.0	0.4037037	0.9814814	72.608
Fine Strut 2.5	0.396296	1.318519	79.665
Mid Strut 2.0	0.407407	0.944444	65.083
Mid Strut 2.5	0.407407	1.292593	74.461

As can be seen from Table 2, in both the series of fine strut and the series of mid strut, the projected aperture and porosity increase with the increase of cell size L. The aperture of Fine Strut 2.0 is similar to that of Mid Strut 2.0, and the aperture of Fine Strut 2.5 is similar to that of Mid Strut 2.5.The porosity of Fine Strut 1.75 is similar to that of Mid Strut 2.0, and the porosity of Fine Strut 2.0 is similar to that of Mid Strut 2.5. The porosity of Fine Strut 2.5 is the largest.

**Fig. 2:**
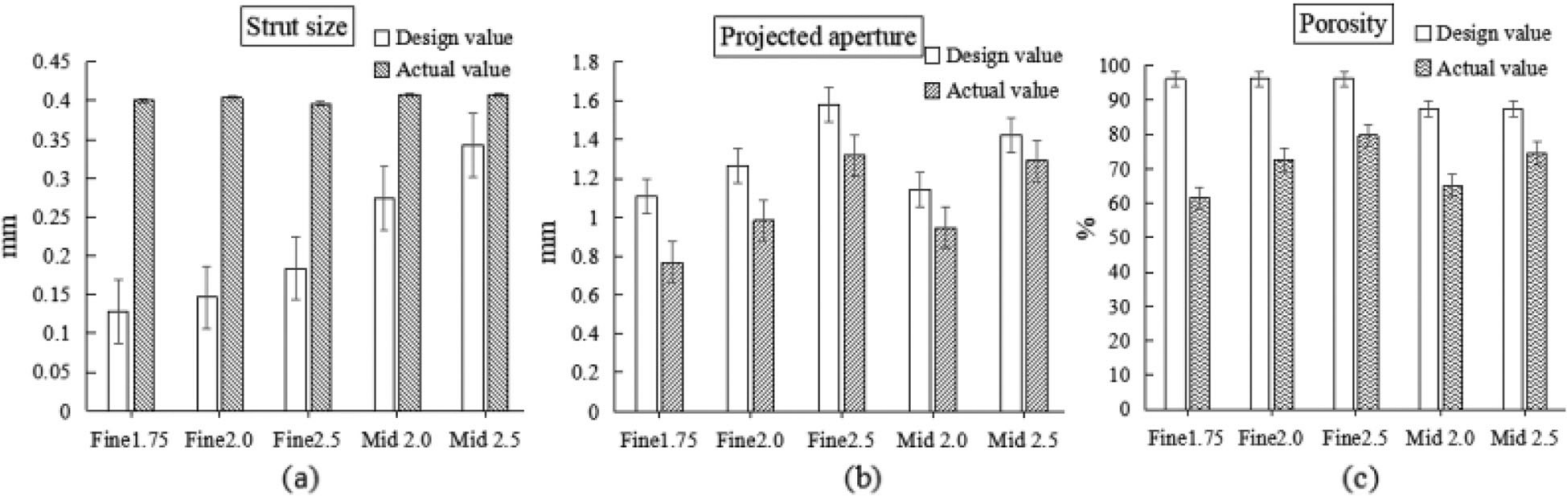
Comparison between design value and actual value of scaffold specimen. (**a**) Strut size, (**b**) Projected aperture, (**c**) Porosity

Figure 2 visually shows the difference between the design value and the actual value of the scaffold specimen. It can be seen that the porosity produced by 3D printing is always lower than that designed.

### Mechanical properties

**Fig. 3:**
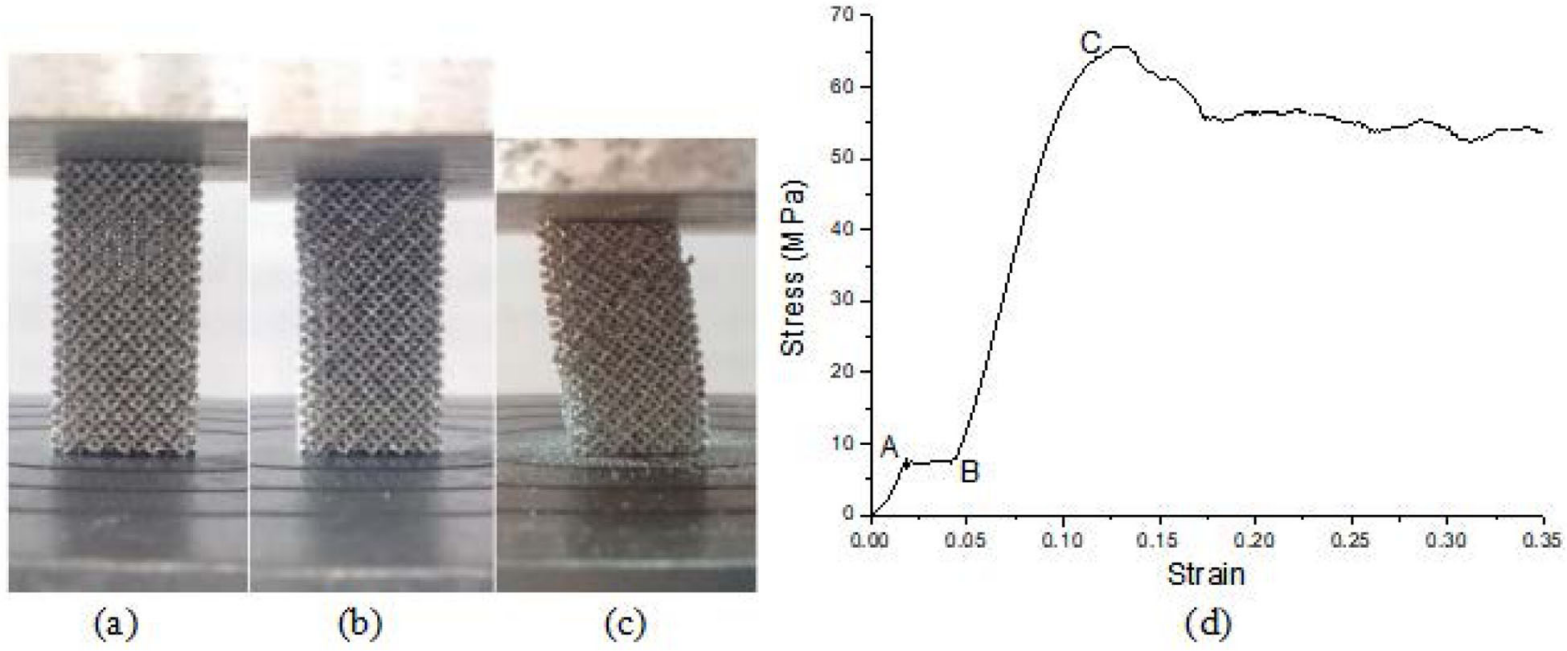
The morphology of the compression process and the compressive stress-strain curve of 3D printed titanium alloy scaffold specimens. (**a**) before compression, (**b**) during compression, (**c**) after compression, (**d**) the compressive stress-strain curve

The morphology of the compression process is shown in Fig. 3a-c.

**Fig. 4:**
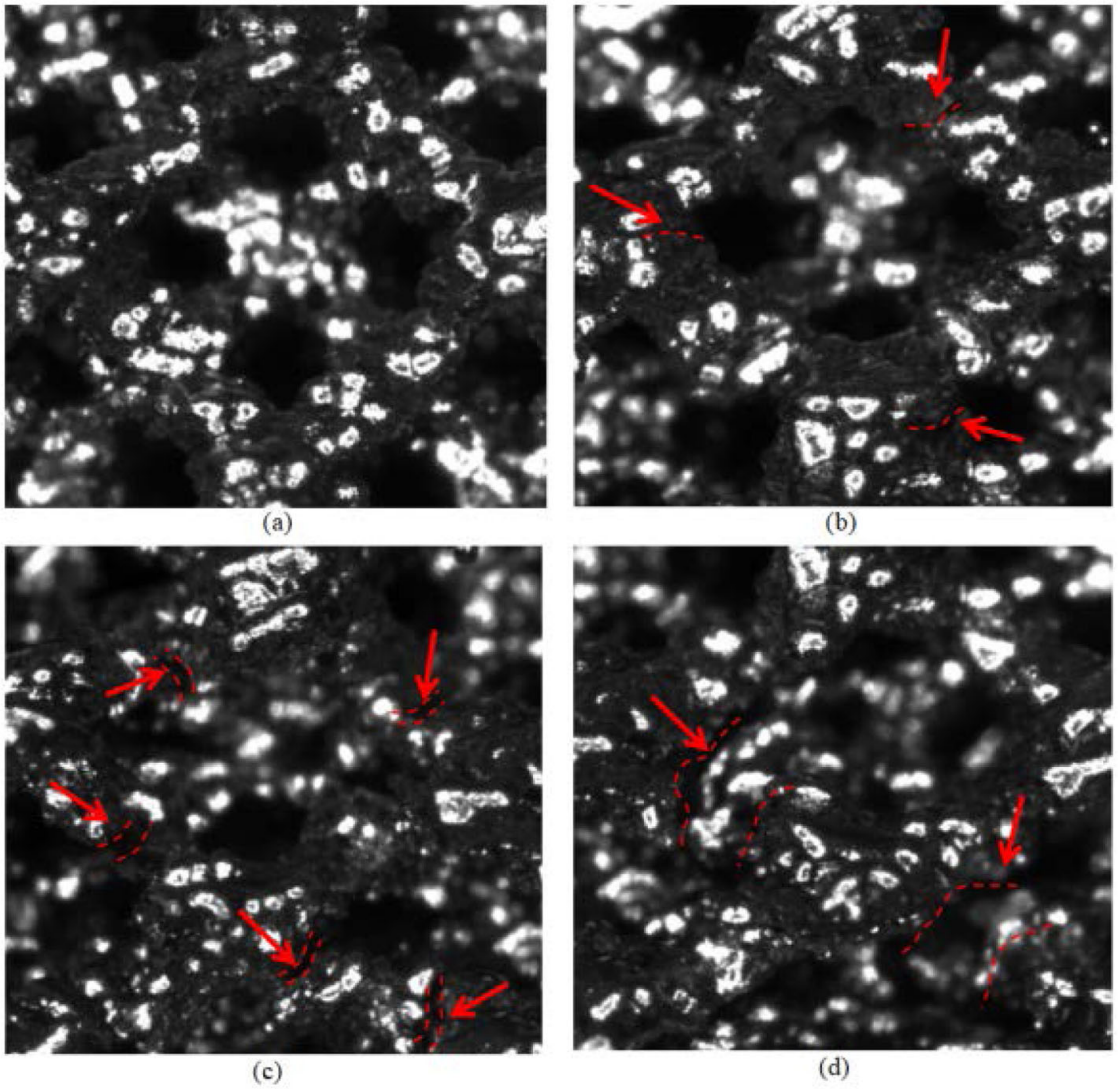
Crack initiation and propagation during compression process (the red dotted line in the figure represents the crack). (**a**) the first stage, (**b**) the second stage, (**c**) the third stage, (**d**) the fourth stage

As shown in Fig. 3d, the compression stress-strain curves of 3D printed titanium scaffold specimens are divided into four stages. The first three stages had the three stage characteristic of the typical stress-strain curve of the foam metal compressed [30], that is, the obvious linear elastic deformation stage OA, the yield plateau stage AB and the compact stage BC. In the first stage, that the linear elastic deformation stage OA, under compressive loading, struts of cells of scaffold structure first undergoes micro-bending (Fig. 4a). After reaching the critical stress, it enters the second stage, that the yield plateau stage AB. At this stage, cells’ struts further deforms. Firstly, microcracks appear at the corner of the cell structure, and the original diamond-shaped pores becomes flattened (Fig. 4b). At this time, the internal stress does not continue to increase but the strain continues to increase, that is, a stress platform appears and the specimen undergoes plastic deformation, which is due to the plastic hinge occurring at the maximum bending moment of metal bone trabecula, which is the elastic-plastic body. Therefore, the continuous strain behavior of the metal bone trabecula near a fixed stress value is shown as the yield platform stage on the stress-strain curve. Its deformation cannot be restored. When the load on the specimens reaches the yield limit, it enters the third stage, that the compact stage BC. There are many cracks breeds on cells’ struts, and the cracks on the corner become larger or even break (Fig. 4c). From the macroscopic point of view, it can be seen that some of the struts are twisted, but the failure of the specimens is not obvious (Fig. 3b). In the fourth stage, that the crushing stage C, some cells’ struts are broken and collapsed (Fig. 4d).

#### The effects of different aperture and porosity on mechanical properties

**Fig. 5:**
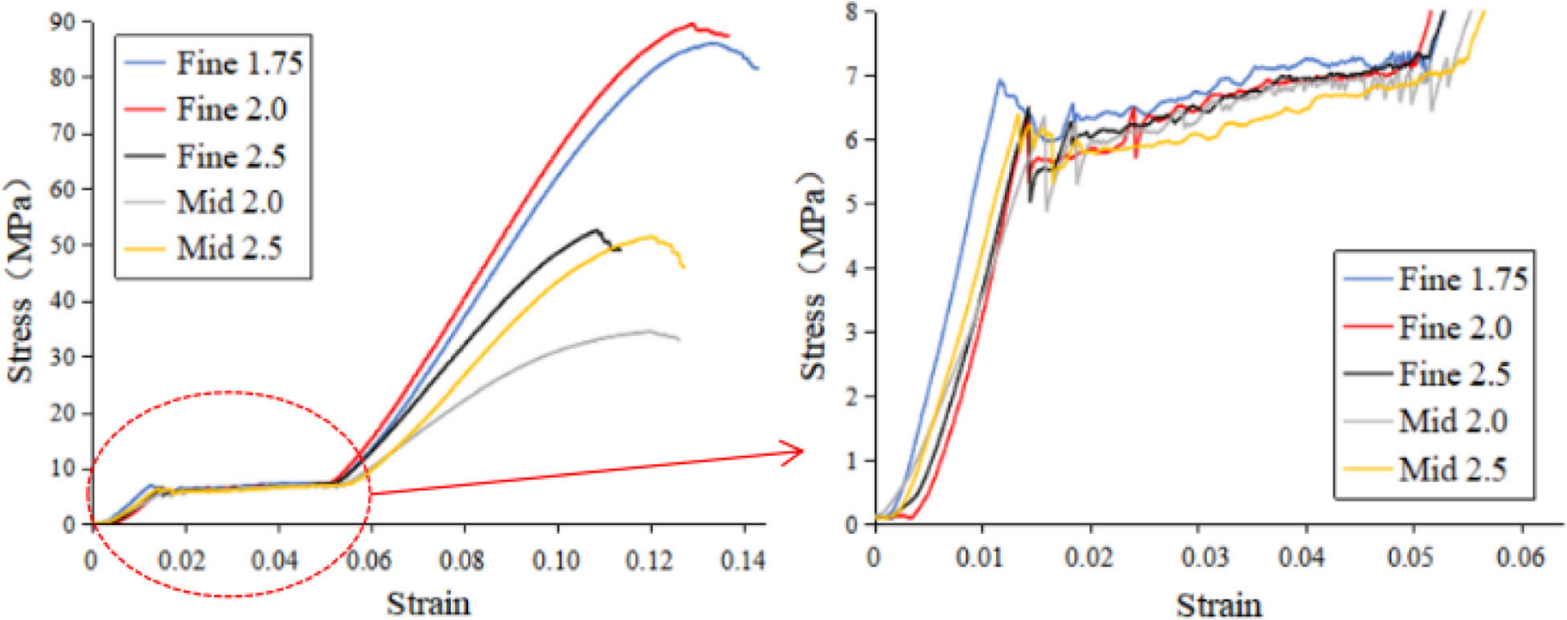
Compressive stress-strain curves and local enlargement maps of scaffold specimens of different specifications

Compressive stress-strain curves of scaffold specimens of different specifications are shown in Fig. 5. It can be clearly seen from it that the load-carrying capacity changes with the increase of cell size L, in both the series of fine strut and the series of mid strut. The load-carrying capacity of Fine Strut 1.75 is similar to that of Fine Strut 2.0, and the load-carrying capacity of Fine Strut 2.5 is similar to that of Mid Strut 2.5. The bearing capacity of Mid Strut 2.0 is the worst.

The compression performance of the structure is shown in Table 3. It can be seen from the table that the maximum force, compressive strength, yield force, yield strength and modulus of elasticity (stiffness) change with the increase of cell size L in both the series of fine strut and the series of mid strut. Its structural stiffness ranges from 437 to 659 MPa, and the corresponding compressive strength ranges from 30.27 to 80.34 MPa.

**Table 3:** Compression experimental data of scaffold specimens

Specimen specifications	Maximum Force (N)	Compressive Strength (MPa)	Yield Force (N)	Yield Strength (MPa)	Modulus of Elasticity (Mpa)
Fine Strut 1.75	8480.09	77.07	8405.78	76.39	659.33
Fine Strut 2.0	8717.44	80.34	8632.83	79.44	628.78
Fine Strut 2.5	4724.88	43.01	4444.3	40.59	548.69
Mid Strut 2.0	3421.05	30.27	3254.2	32.32	437.09
Mid Strut 2.5	5081.16	47.06	5004.23	46.34	545.22

#### Anisotropy of structure

**Fig. 6:**
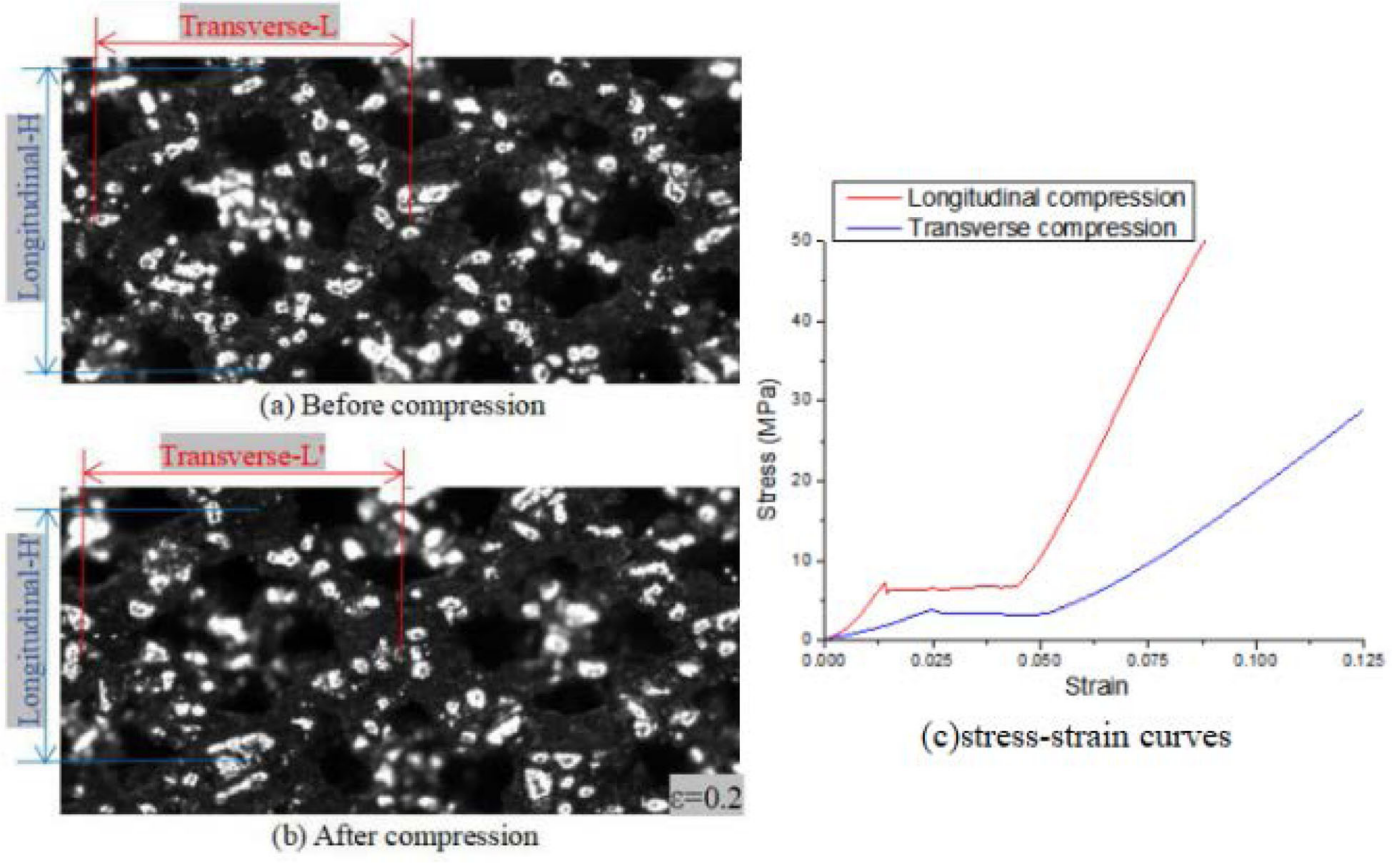
Calculating Poisson ratio of this structures and the comparison of stress-strain curves between longitudinal and transverse compression. (**a**) before compression, (**b**) after compression, (**c**) the compressive stress-strain curve

Taking scaffold specimens whose specifications are “Fine strut 2.0” to implement longitudinal compression and transverse compression respectively, the scaffold structure shown the anisotropy. The stress-strain curves of longitudinal and transverse compression (Fig. 6c) are similar in shape but do not coincide. From the elastic stage, the elastic modulus of longitudinal compression is obviously higher than that of transverse compression. Therefore, the more the number of cells parallel to the compressive load, the greater the load-carrying capacity of the scaffold structure is.

Measurement by digital image correlation equipment (as shown in Fig. 6a-b), the Poisson’s ratio of the trabecular structure was calculated to be 1.23, as shown in Table 4.

**Table 4:** Measured data and calculation of Poisson ratio of this scaffolds

	Transverse dimension (mm)	Longitudinal dimension (mm)	Transverse strain	Longitudinal strain	Poisson ratio
Before compression	L = 0.1589	H = 0.1578	0.9717	0.7883	1.23
After compression(ε = 0.2)	L’ = 0.1544	H′ = 0.1244			

#### The effects of different loading speeds on mechanical properties

**Fig. 7:**
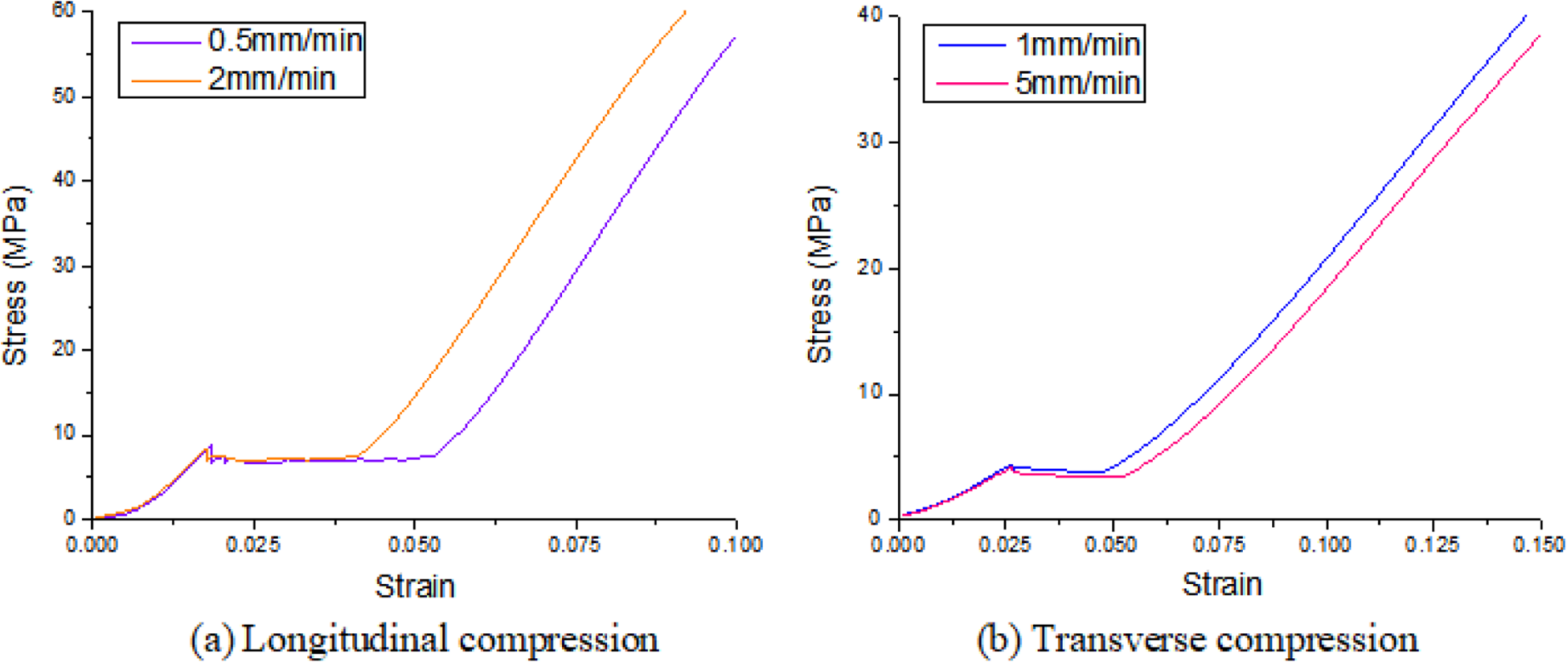
The comparison of compressive stress-strain curves at different loading speeds. (**a**) longitudinal compression, (**b**) transverse compression

When the scaffold of “Fine Strut 2.0” is compressed longitudinally, the stress-strain curve obtained at the loading speed of 0.5 mm/min and 2 mm/min is shown in Fig. 7a, and the stress-strain curve obtained at the loading speed of 1 mm/min and 5 mm/min is shown in Fig. 7b. It can be seen that the stress-strain curves of two specimens with the same specification are similar under two different loading speeds, which indicates that the loading speed has little effects on the mechanical properties of the specimens under quasi-static loading conditions.

#### The differences in mechanical properties of specimens taken from different directions and sites of 3D printers’working space

A batch of scaffold specimens whose specifications are “Fine strut 2.0” were manufactured by 3D printing, which were taken from nine locations in the 3D printer’s workspace, respectively. The nine directions are: ZQS, ZQX, ZHS, ZHX, ZJ, YQS, YQX, YHS and YHX. There are three specimens in each direction, a total of 27 specimens. The effect of different printer locations on mechanical properties of specimens was studied.

**Fig. 8:**
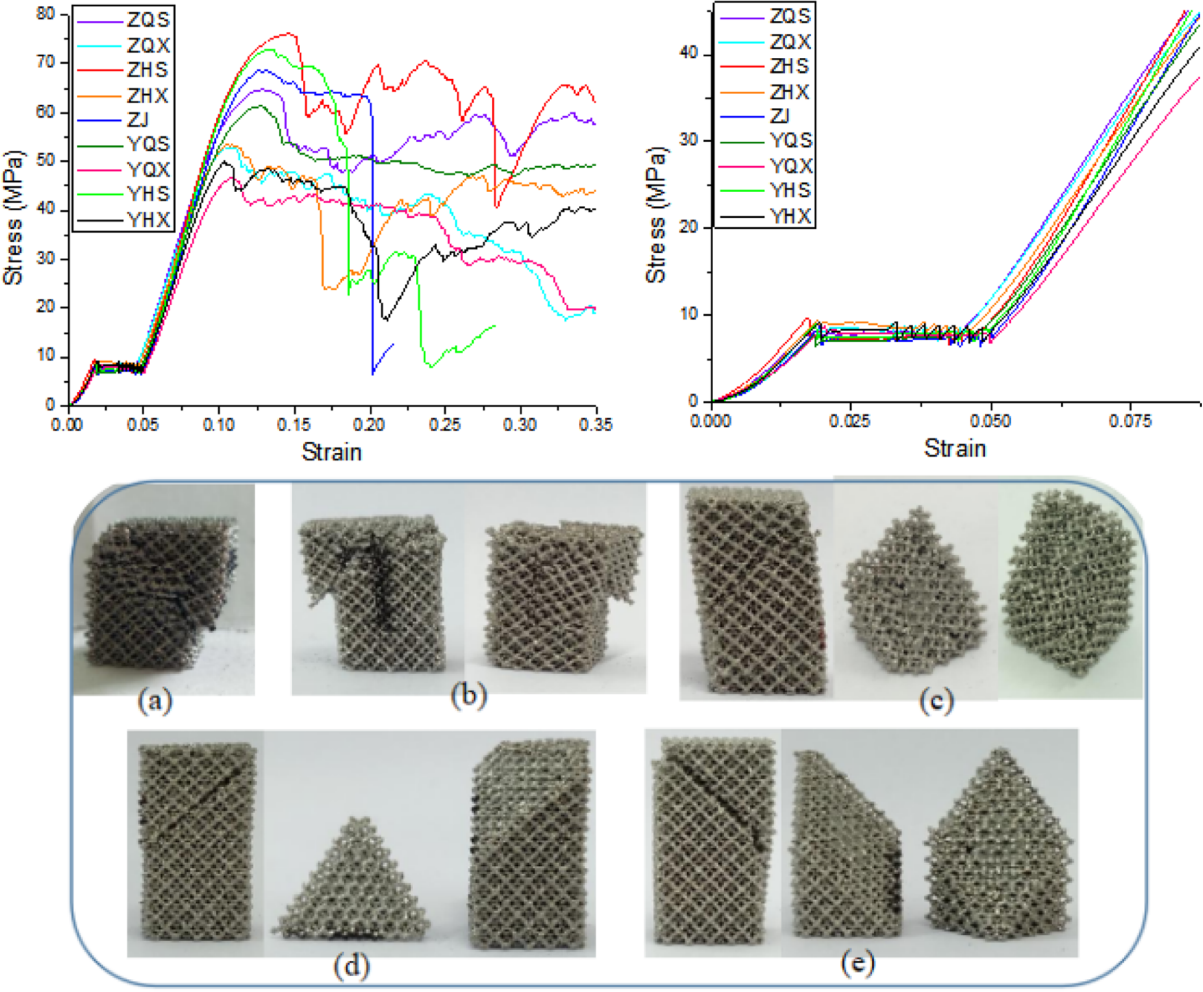
Compressive stress-strain curves of 9 specimens from 9 different locations and their local magnification maps, and fracture morphology of some specimens. the (**a**) to (**e**) are the fracture patterns of five specimens

The compressive stress-strain curves with the same loading speed of 9 specimens whose specifications are “Fine strut 2.0” from 9 locations of 3D printer and their local magnification figures are shown in Fig. 8. The stress-strain curves of the specimens printed on the nine directions of the 3D printer are similar in shape and have little difference, which indicates that the different directions of the 3D printer have little effect on the mechanical properties of the specimens.

### Failure mechanism

It was found by experiment that, whether longitudinal or lateral compression, the failure is mainly caused by shear stress of 45 degrees.

The curve shape of crushing stage, namely the fourth stage of the stress-strain curve (as shown in Fig. 8), can be roughly summarized into the following four types. The first one, if struts of cells is broken evenly and no large block collapses during compression, there is a long microwavy stress “platform” shown as Fig. 8-YQS. After compression, the specimen is relatively complete and the damaged part still stick to the main block (Fig. 8a). The second one as morphology of the specimen after compression shown in Fig. 8b, if a small part of cells structure is broken but not collapse and completely disconnected from the whole, the stress increases gradually and tends to be wavy “platform” as shown in Fig. 8-ZHX. The third one, if the structure breaks entirely during compression, the stress decreases as microwave fluctuation (Fig. 8-ZQX). The overall fracture surface is 45^o^ inclined to the loading direction, while, the inclined plane is uneven (Fig. 8c), which is the most common situation appeared in the experiments. The last one, if brittle fracture occurs the stress drops sharply (Fig. 8-ZJ). The whole fracture surface is 45^o^ inclined and the inclined plane is very smooth (Fig. 8d-e).

## Discussion

We systematically studied a 3D printed titanium alloy scaffold mimicking trabecular structure, from its structural design, fabrication to the mechanical behavior. At present, the structures and functions are the focus of clinical application. The porosity of scaffold manufactured by EBM is lower than that of design. The bearing capacity of scaffold is related to the porosity of the structure. The fracture interface of the scaffold is 45^o^ inclined to the loading direction, which conforms to Tresca yield criterion. The scaffold mimicking trabecular structure are anisotropic. The loading speed has no effect on the mechanical properties of the specimens under quasi-static loading conditions. Structural morphology is related to the bioactivity of implants. The proper porosity and connectivity are conducive to the adhesion, proliferation and differentiation of osteoblasts, and can guide bone tissue to grow into the pore. Mechanical behavior is related to the effectiveness of mechanical transmission of prostheses.

Compared with 3D printed dense titanium alloy materials, the elastic modulus and strength of 3D printed titanium alloy bone trabeculae are greatly reduced.

Tensile specimens of 3D printed dense titanium alloy were tested. After 3D printing, heat treatment was carried out to eliminate prestressing force. There were a total of 18 specimens distributed in 9 different locations on the 3D printer’workspace. The specimen is cylindrical with cross-sectional diameter of 5 mm and length of 25 mm respectively. The stress-strain of specimens curve is obtained by tensile test. The tensile test data are shown in Table 5.

**Table 5:** Result data of Tensile Tests of 3D printed titanium alloy dense specimens (Data Average of 18 Specimens)

Maximum force (N)	Tensile strength (MPa)	Lower yield force (N)	Lower yield strength (MPa)	Elongation at break (%)	Section shrinkage (%)	Modulus of elasticity (MPa)
20,137.5	1014.3	19,457.6	980.1	13.12	29.84	10,088.49

Tensile tests of 3D printed Titanium alloy dense specimens show that there are obvious yielding and necking phenomena, as displayed in plastic materials. The strength of the material is higher, approaching 1GPa, however, the elastic modulus is lower with the average value of 10GPa, which is close to the mechanical properties of forged titanium alloy (1000 MPa/110GPa). Compared with the high strength and low elastic modulus of 3D printed dense titanium alloy specimens, the elastic modulus and strength of 3D printed porous titanium alloy decreases by 96% - 93% and 96% - 91% respectively. The apparent modulus of elasticity is 0.39–0.618 GPa, which indicates that the mechanical propertie of bone trabeculae with this porous structure are closer to that of human bone, and stress shielding can be reduced effectively.

This paper systematically correlates the scaffold structure to mechanical behavior of a 3D printed titanium alloy scaffold, aiming at mimicking the mechanical properties of natural trabecula for tissue engineering. However, it is more or less ignored that the biological and physiological function of the osteoblast seeded in the titanium alloy porous scaffold and the interaction between cells and surface, which will be the future focus in the following study.

## Conclusion

Titanium alloy is widely used in the field of clinical bone implants due to its excellent biocompatibility, biological safety and mechanical strength. It is an ideal material for the production of artificial joint prosthesis and bone joint products. The parameters of 3D printed titanium alloy scaffold, such as aperture, porosity, pore shape and surface treatment, have an important effect on bone growth and osteoblast performance in vitro. We have systematically studied the structural design, fabrication and mechanical behavior of a 3D printed titanium alloy scaffold mimicking trabecula bone. This study may shed a light on the application of prostheses with proper structures and functions.

## Data Availability

Data and materials presented in this paper can be shared upon request.

## Abbreviation

EBM: Electron beam melting
